# Dynamic stability requirements during gait and standing exergames on the wii fit® system in the elderly

**DOI:** 10.1186/1743-0003-9-28

**Published:** 2012-05-20

**Authors:** Cyril Duclos, Carole Miéville, Dany Gagnon, Catherine Leclerc

**Affiliations:** 1 Pathokinesiology laboratory, Centre for Interdisciplinary Research in Rehabilitation (CRIR), Institut de réadaptation Gingras-Lindsay-de-Montréal (IRGLM), 6300 avenue Darlington, Montréal, QC, H3S 2 J4, Canada; 2 School of Rehabilitation, Université de Montreal, 7077, avenue du Parc, Montréal, QC, H3N 1X7, Canada

**Keywords:** Equilibrium, Dynamic balance, Biomechanics, Aging, Human

## Abstract

****Background**:**

In rehabilitation, training intensity is usually adapted to optimize the trained system to attain better performance (overload principle). However, in balance rehabilitation, the level of intensity required during training exercises to optimize improvement in balance has rarely been studied, probably due to the difficulty in quantifying the stability level during these exercises. The goal of the present study was to test whether the stabilizing/destabilizing forces model could be used to analyze how stability is challenged during several exergames, that are more and more used in balance rehabilitation, and a dynamic functional task, such as gait.

****Methods**:**

Seven healthy older adults were evaluated with three-dimensional motion analysis during gait at natural and fast speed, and during three balance exergames (50/50 Challenge, Ski Slalom and Soccer). Mean and extreme values for stabilizing force, destabilizing force and the ratio of the two forces (stability index) were computed from kinematic and kinetic data to determine the mean and least level of dynamic, postural and overall balance stability, respectively.

****Results**:**

Mean postural stability was lower (lower mean destabilizing force) during the 50/50 Challenge game than during all the other tasks, but peak postural instability moments were less challenging during this game than during any of the other tasks, as shown by the minimum destabilizing force values. Dynamic stability was progressively more challenged (higher mean and maximum stabilizing force) from the 50/50 Challenge to the Soccer and Slalom games, to the natural gait speed task and to the fast gait speed task, increasing the overall stability difficulty (mean and minimum stability index) in the same manner.

****Conclusions**:**

The stabilizing/destabilizing forces model can be used to rate the level of balance requirements during different tasks such as gait or exergames. The results of our study showed that postural stability did not differ much between the evaluated tasks (except for the 50/50 Challenge), compared to dynamic stability, which was significantly less challenged during the games than during the functional tasks. Games with greater centre of mass displacements and changes in the base of support are likely to stimulate balance control enough to see improvements in balance during dynamic functional tasks, and could be tested in pathological populations with the approach used here.

## Introduction

Balance exercises are a key component of multifactorial fall prevention programs in elderly individuals [1] or persons with hemiparesis [2]. However, the choice of balance exercises included in these training programs is rarely discussed in the literature. Moreover, no training principle has been proposed to ensure the effectiveness of these balance exercises. Contrary to resistance training where precise training parameters have been determined [3], the progressive overload principle that stipulates to work at a relative level of maximum capacity, known to be essential for the system to adapt to the new level of constraint [3], has not been tested or adapted for balance training to date.

Mansfield et al. recently proposed to apply the overload principle in a perturbation-based balance training program [4]. These external perturbations, delivered by a moving platform on which the participants were standing, were considered an overload since this type of difficulty is rarely encountered in daily living, and were gradually intensified by increasing the amplitude of the perturbation. This program was shown to improve the postural reactions of participants with a recent history of a fall in terms of the frequency of multiple-step reactions or time to reach the handrail. No statistics were obtained on the impact of the program on functional capacity, fall rate or circumstances after training [5]. It seems necessary to question whether the proposed level and type of overload is appropriate for improving balance in daily life among participants. In addition to these so-called external perturbations, self-induced disequilibrium is also known to cause falls in older persons during activities [6-9]. Self-induced perturbations are loss of balance due to the person's own movements. Thus, training sessions must also match the characteristics of normal functional activities in terms of movement strategies (specificity principle), i.e. postural adjustments to reduce loss of balance due to the displacements of the centre of mass during normal activities. In terms of intensity, the balance exercises should exceed the usual postural difficulty level encountered in most daily activities (overload principle) to improve or maintain balance abilities for most everyday situations if those principles also apply in balance rehabilitation.

To ensure that the level of stimulation of the balance system is adequate to improve balance, it would be beneficial to grade the intensity level of the balance exercises based on the abilities and goals of the subjects. We propose to use a recently developed biomechanical model to evaluate stability during functional tasks [10] for that purpose. The model is based on two complementary concepts: 1) *destabilizing force*, that is, the theoretical force necessary, if applied to the body, to bring the body into an unstable state, or, in other words, the force necessary for the centre of mass and centre of pressure to reach and stop at the limit of the base of support, i.e. with no velocity at that point; and 2) *stabilizing force*, that is, the theoretical force needed to stop the displacements of the centre of mass and centre of pressure at the limit of the base of support. These two forces are measured at each moment of the task using three-dimensional biomechanical analysis of the body movements and measurement of ground reaction forces (see [10] for details). The destabilizing force mainly evaluates the postural aspect of balance, i.e. how the body is placed over the base of support (also called postural orientation) and the stabilizing force primarily evaluates the dynamic aspect of balance [11-13], i.e. how displacement of body mass puts stability at risk (also called equilibrium [12]). The ratio between the destabilizing and stabilizing forces results in an index of stability. A higher stabilizing force value combined with a lower destabilizing force value generates a low stability index, which reflects low dynamic stability.

This model will be used with persons who are playing exergames from a commercially available low-cost virtual reality system. This type of system has recently generated great interest from clinicians, partly due to the motivational aspect of this low-level virtual reality system [14-17], even though the effectiveness of visual feedback, one of the components often used as a simpler version of virtual reality in rehabilitation, is still under debate [18,19]. The use of visual feedback or virtual reality in balance training has already been evaluated following stroke, with very limited functional results according to systematic reviews [20-22]. A recent randomized, controlled trial expanded on and confirmed the results of these systematic reviews. This study demonstrated an increase in endurance (measured using the 6-min Walk Test) but yielded limited results for the balance tests in the group with visual feedback training compared to the traditional balance training group, which saw significant improvements in their scores in the modified clinical test of sensory interaction and balance [23]. The proposed balance exercises did not require body displacements beyond the shifting of weight between both feet, and may have stimulated dynamic stability at a low level.

The present study was designed to determine whether the model can show differences in how three exergames challenge the stability of older subjects compared to natural and fast gait speed. Global, postural and dynamic aspects of balance were evaluated during these five tasks by means of the stability index and destabilizing and stabilizing forces, respectively. It was hypothesized that stability would be lower during the gait tasks due to the greater displacements of the centre of mass, resulting in greater dynamic instability, and due to the centre of pressure being positioned closer to the limit of the base of support, resulting in greater postural instability.

## Methods

### Subjects

A convenient sample of seven (two men, five women) healthy older participants (mean (SD) age: 66 years, 7 months, (4 years, 5 months); mean weight: 74.6 (11.5) kg; mean height: 1.65 (0.08) m) attended the clinical and laboratory evaluations after providing informed consent, in accordance with the rules set out by the Research Ethics Committee of the Centre for Interdisciplinary Research in Rehabilitation of Greater Montreal. The group had a good level of balance and physical abilities according to the following clinical tests: the Berg Balance Scale (56/56 [53;56] (median score [range])), the Timed-Up-and-Go Test (9.1 (1.0) s (mean (SD)) at natural speed; 7.0 (1.1) s at fast speed), the 5-Stand-Up-Test (10.2 (2.1) s). The mean walking speed was 1.4 (0.2) m/s at natural and 1.9 (0.3) m/s at maximal speed, as measured over a 10-m distance. All these tests have good psychometric properties in older adults [24-28].

### Stability evaluation

A stability assessment was conducted in a motion analysis laboratory. Kinematic data were recorded at 60 Hz using an Optotrak 3020 system (Northern Digital Inc.) and 36 infrared markers placed over the entire body. Three to four non-collinear markers were placed on the following body segments: feet, legs, thighs, pelvis and trunk. Two markers on the head and on the C7 vertebrae were used for the head; one marker was placed at the shoulder, elbow and wrist joint axis for the upper limbs. Specific anatomical points were probed to determine their position according to the markers on their respective body segment. The contour of each foot was probed to define the base of support according to the position of the feet. Anthropometric data were measured clinically for each segment to define rigid bodies in three dimensions from markers and probed anatomical points, as well as the position of the centre of mass and the radii of gyration according to regression equations [29]. Ground reaction forces were measured at 600 Hz using AMTI force plates (OR-6-5-1) embedded in the floor. The data were filtered with a fourth order Butterworth zero-lag filter, with a cut-off frequency of 10 Hz and then down-sampled at 60 Hz to match the kinematic data.

Five tasks were evaluated: natural and fast gait speed, and three games from the balance training section of the Nintendo Wii Fit® system, with an a priori increasing level of difficulty. The first game was the 50/50 Challenge, in which the player has to maintain equal body weight between the two lower limbs, using visual feedback of weight distribution and the target. The data were obtained at the highest level of difficulty reached by the participant (narrowest target around the 50% goal). The second game was the Ski Slalom during which the player has to shift his weight from one leg to the other to control the avatar and lead it between slalom gates. The last game was Soccer, during which the player also has to shift his weight from one leg to the other to move the avatar’s body and head in front of a ball coming at the avatar. In this last game, catch trials, i.e. something other than a ball is kicked at the player, reduced the possibility for the player to anticipate, contrary to the Slalom game, in which the path to the gates could be anticipated. The final scores of the players were not taken into account, as only the stability requirements during the tasks were analyzed, without any focus on the level of performance in the game. As for visual feedback, a 21-inch TV monitor was placed 160 cm in front of the Wii Fit® platform, 80 cm above the ground. The order of the tasks was pseudo-randomized, with the two gait tasks performed first or last to simplify experimental set-up. Practice trials were allowed until the principle of the game was understood and the participants were comfortable with the game. Three 15-s periods for each game and five gait trials at a natural and fast speed, with two consecutive steps, i.e., complete contact of each foot on two different force plates, were recorded. Gait speed was controlled to ensure limited variability for each subject.

### Variables and data analysis

The destabilizing and stabilizing forces as well as the stability index were calculated from the displacements of the centre of mass, centre of pressure and limit of the base of support [10]. The calculation was slightly modified compared to the previously published equations, as the distances used in the equations were both measured between the current position of the centre of pressure (instead of the centre of mass for the stabilizing force) and the point on the limit of the base of support in the direction of the centre of mass displacement. The limit of the potential base of support is still defined as the outside perimeter of the vertical projection of both feet on the floor.

The scores obtained from the clinical evaluation of balance were presented using descriptive statistics. Mean values for both forces and the stability index, as well as the maximum stabilizing force value, minimum destabilizing force value and stability index value were averaged between subjects, and then compared for all five tasks using repeated measures ANOVA. The mean values of the three variables represent the overall stability requirement for each task, while extreme values represent the highest stability requirements during the tasks. The fast gait speed task was considered to represent the subject’s maximum ability. A priori contrasts were planned based on our hypothesis that stability increases between gait at fast speed, gait at natural speed, Soccer, Slalom and 50/50 Challenge. The four planned contrasts were thus gait at fast speed vs. gait at natural speed, gait at natural speed vs. Soccer, Soccer vs. Slalom and Slalom vs. 50/50 Challenge. The three exergames and natural gait speed were then rated against the fast gait speed by calculating the ratios of each value for each task for the same value during the fast gait speed. Because minimum values are associated with instability for the stability index and the stabilizing force, the ratio was calculated as follows: (value at max gait speed/value for each task) *100 for the mean and minimum value of these two variables, and (value for each task/value at max gait speed) *100 for the destabilizing force. In case of rejection of the hypothesis of normal distribution of the data (Kolmogorov-Smirnov test), a non-parametrical approach would be applied. Statistical analysis was performed using SPSS 17.0.

## Results

**Figure 1:**
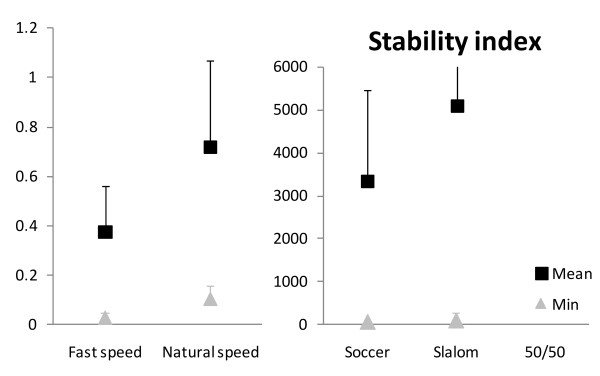
**Mean (black squares) and minimum (grey triangles) stability index values for the group, for the five tasks.** Lower stability index represents lower overall stability. Error bars represent one standard deviation (SD) of the value. The maximums of the vertical axes were chosen to show most of the values despite large scale differences, without flattening the results with lower values. However, the values for the 50/50 Challenge are missing (Min (SD): 10513.8 N (12764.6), Mean (SD): 386783.1 (4.1 x 10^5^)), as well as the SD for Slalom (SD = 2131.2).

**Figure 2:**
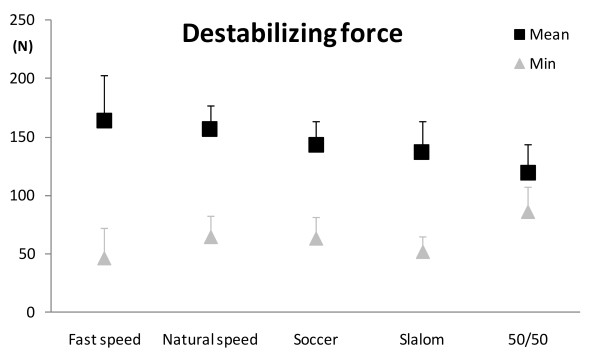
**Mean (black squares) and minimum (grey triangles) destabilizing force values for the group, for the five tasks (N).** Lower destabilizing force represents lower postural stability. Error bars represent one standard deviation (SD) of the value.

**Figure 3:**
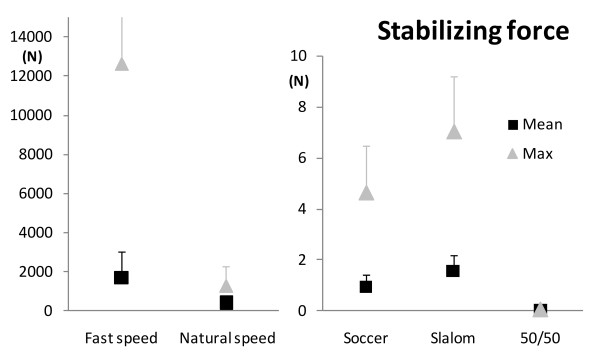
**Mean (black squares) and maximum (grey triangles) stabilizing force values for the group, for the five tasks.** Higher stabilizing force represents lower dynamic stability. Error bars represent one standard deviation (SD) of the value. The maximum of the vertical axes was chosen to show most of the values despite large scale differences, without flattening the results with lower values. The standard deviation for the gait at fast speed is missing (SD = 17334.2 N).

Kolmogorov-Smirnov tests indicated that the data followed a normal distribution for each stability variable (*p* > 0.05). Parametric statistical tests were thus applied. The five tasks had different levels of overall stability requirements, as shown by the mean stability index (repeated measures ANOVA, *p* < 0.05), decreasing from the 50/50 Challenge towards the gait at fast speed (contrasts, *p* < 0.05), except for the Slalom and Soccer exergames which were similar (*p* = 0.63) (Figure 1 and Table 1). The mean destabilizing force also differed between the tasks (repeated measures ANOVA, *p* < 0.05) (Figure 2 and Table 1), with lower value during the 50/50 Challenge than during Slalom (*p* < 0.05) only, and no difference in the three other contrasts (*p* > 0.40). In terms of the stabilizing force, it also differed between the tasks (repeated measures ANOVA, *p* < 0.05), each one being smaller than the next one (contrasts, *p* < 0.05), from gait at fast speed to 50/50 Challenge, except for Soccer and Slalom, which did not differ significantly (*p* = 0.087) (Figure 3 and Table 1).

**Table 1:** Stability variables for the different tasks

		50/50 Challenge	Slalom	Soccer	Natural Gait Speed	Fast Gait Speed
Stability Index	Mean	**386783.1 (4.10**^**5**^**)**	3337.2 (2131.2)	**5089.7 (9591.6)**	**0.72 (0.35)**	0.37 (0.18)
	Min	10513.8 (12764.6)	87.2 (185.9)	**54.8 (40.7)**	**0.10 (0.06)**	0.03 (0.02)
Destabilizing Force (N)	Mean	**119.2 (25.0)**	137.3 (26.7)	143.0 (20.2)	156.04 (39.4)	164.0 (20.4)
	Min	**86.1 (22.0)**	51.9 (12.8)	63.3 (18.0)	**64.6 (25.9)**	46.5 (18.6)
Stabilizing Force (N)	Mean	**0.01 (0.01)**	**1.57 (0.6)**	**0.9 (0.5)**	**420.0 (219.6)**	1711.5 (1314.9)
	Max	**0.05 (0.05)**	7.0 (2.2)	**4.6 (1.8)**	1276.4 (993.2)	12629.0 (17334.2)

Bold indicates statistical difference (*p* < 0.05) with the next task on the right in a priori planned contrasts. Values are indicated as mean (SD).

As for the most instable moment of each task, results for the minimum stability index were similar to the mean stability index (repeated measures ANOVA, *p* < 0.01)(Figure 1, Table 1), with the least stability being lower for gait at fast speed, and increasing at natural speed (*p* < 0.005), and for Soccer (*p* < 0.05). Slalom did not differ from Soccer (*p* = 0.66). 50/50 Challenge did not reach the level of statistically significant difference compared to Soccer (*p* = 0.07). The minimum destabilizing force also differed according to the tasks (repeated measures ANOVA, *p* < 0.001) (Figure 2, Table 1): its value for the 50/50 Challenge was higher (*p* < 0.05) than for the Slalom and at natural speed compared to fast speed (*p* < 0.05). Slalom did not differ from Soccer (*p* = 0.24), which did not differ from gait at natural speed (*p* = 0.89). Finally, the maximum stabilizing force differed between the five tasks (repeated measures ANOVA, *p* < 0.05) (Figure 3, Table 1): it did not differ between natural and fast gait speed (*p* = 0,137), but was smaller during Soccer than gait at natural speed (*p* < 0.05) and during 50/50 Challenge than Slalom (*p* < 0.001). The difference between Soccer and Slalom (*p* = 0.069) did not reach statistical significance.

The ratios calculated to determine the balance requirements of the tasks showed that the level of stimulation is generally low (50% or lower) compared to the fast gait speed except for the destabilizing force, i.e., the postural aspect of balance (see Table 2).

**Table 2:** Grading of balance requirements

		Soccer	Slalom	50/50 Challenge	Natural Gait Speed
Stability Index	Mean	0.0	0.0	0.0	52.4
	Minimum	0.0	0.0	0.0	51.7
Destabilizing Force	Mean	114.6	119.4	137.5	104.8
	Minimum	73.4	89.5	54	72
Stabilizing Force	Mean	0.1	0.1	0.0	24.5
	Maximum	0.0	0.1	0.0	10.1

Grading was calculated for the three exergames (Soccer, Slalom and 50/50 Challenge) as well as natural gait speed versus fast gait speed (expressed as a percentage (%)).

## Discussion

The results of this study confirmed the hypothesis that stability requirements during exergames were much lower than during gait at natural or fast speed, as indicated by the mean stability index of each task. These exergames demanded body weight transfers, or standing with equal weight distribution between both feet. The last task, i.e. the 50/50 Challenge, clearly differed from the other tasks with a very high mean stability index value. This was expected due to the obviously limited displacements of the body during the task. This was evidenced by the very low stabilizing force value (0.01 N), indicating that the difficulty to stop the displacements of the body is very low. This difficulty was higher for the two other games, with weight transfers between both feet, but still did not reach the level of stabilizing force computed during natural gait or even fast gait. Mean and extreme stabilizing force and index followed the same pattern, with the largest dynamic instability and overall instability, respectively, for gait at fast speed, and the smallest for the 50/50 Challenge. Thus, exergames did not challenge the dynamic aspect of balance very much during the entire task or at the highest instability point of the task, compared to the balance requirements encountered during gait.

Surprisingly, the mean destabilizing force during the 50/50 Challenge was lower compared to the other tasks, indicating that it was, on average, easier to bring the body into an unstable position during this task than during the other ones. This is likely due to a smaller distance between the centre of pressure and the limit of the base of support during the 50/50 Challenge. In this game, the centre of pressure was centred in the base of support. In the other games where weight shifts were required, the centre of pressure was closer to the limit of the base of support from time to time but the direction of the centre of mass displacements, in which the distance between the centre of pressure and the limit of the base of support is measured, made this distance larger. A larger distance between the centre of pressure and the limit of the base of support increased the average postural stability, as measured by the model in the other games. The results for the minimum destabilizing force value confirmed this, as this value was higher in the 50/50 Challenge than for all the other tasks. This indicates that despite higher mean body posture instability during the 50/50 Challenge, the body was placed in a more challenging position at least once (much more according to the visual analysis of the destabilizing force) during the other games. Higher postural requirements were particularly present before changing direction during body weight shifts.

A weakness of the study is that the results have restricted generalizability because of the limited number of participants. Thus, the results mainly indicate that the games evaluated are possibly of little help to overcome the reduced dynamic control of balance capabilities in the tested group. However, they also suggest they could be useful in populations with static balance difficulties such as persons with amputation or hemiparesis, who often present asymmetrical standing balance. Further analysis in these populations is warranted, and also in healthy older persons with balance difficulties. On the other hand, the results corroborate the few studies that showed improvements mainly in static balance tasks using the same system or weight shifting exercises [20-22]. Further studies are necessary to test the level of balance requirement necessary in balance exercises to achieve good balance improvements, and to determine if there are better tasks than gait at fast speed to be considered maximum abilities of the subjects. Finally, this preliminary study confirms that the stabilizing/destabilizing force model is sensitive to different levels of stability, and powerful enough to show differences in stability requirements even in a small group of healthy participants. The combined analysis of mean and extreme values of the two forces and the index was useful in analyzing the actual level of challenge, but other variables extracted from the data could benefit from being tested further in the future. An analysis of the variability of the results over several successive steps will also be explored as it is well known that balance is controlled over several steps during gait. This was not possible in the present experimental setting. The model could thus help to determine how exercises challenge postural control and testing if the overload principle applies to balance training.

To conclude, use of the stabilizing/destabilizing force model showed that the level of challenge of balance during different exergames was similar to gait at natural speed for static, i.e. postural, aspect of balance. However, the level of challenge was poor for its dynamic aspect compared to gait at natural or fast speed. Thus, grading dynamic and postural stability requirements of balance exercises with respect to functional tasks is feasible and desirable to organize proper progression and potentially sufficient difficulty level to train balance in the most efficient manner, depending on the abilities and functional goals of the participant. Further study is warranted to test the impact of various levels of challenge for postural control during exercises on the rate of balance ability improvements in different pathological populations. More dynamic exergames also need to be tested, with greater centre of mass displacements and base of support configuration changes to reach stability requirements closer to, or even beyond, major functional tasks.

## Competing interests

The authors declare they have no financial or non-financial competing interests.

## Authors’ contributions

CD conceived of the study, its design, coordinated and drafted the manuscript. CM and DG critically revised the manuscript. CL made substantial contributions to acquisition of data, data analysis and critically revised the manuscript. All authors read and approved the final manuscript.
